# Evaluating a radiotherapy deep learning synthetic CT algorithm for PET-MR attenuation correction in the pelvis

**DOI:** 10.1186/s40658-024-00617-3

**Published:** 2024-01-29

**Authors:** Jonathan J. Wyatt, Sandeep Kaushik, Cristina Cozzini, Rachel A. Pearson, George Petrides, Florian Wiesinger, Hazel M. McCallum, Ross J. Maxwell

**Affiliations:** 1 Translation and Clinical Research Institute, Newcastle University, Newcastle upon Tyne, UK; 2 Northern Centre for Cancer Care, Newcastle upon Tyne Hospitals NHS Foundation Trust, Newcastle upon Tyne, UK; 3 GE Healthcare, Munich, Germany; 4 Department of Quantitative Biomedicine, University of Zurich, Zurich, Switzerland; 5 Nuclear Medicine Department, Newcastle upon Tyne Hospitals NHS Foundation Trust, Newcastle upon Tyne, UK

**Keywords:** Synthetic CT, PET-MR, Attenuation correction, Radiotherapy

## Abstract

**Background:**

Positron emission tomography–magnetic resonance (PET-MR) attenuation correction is challenging because the MR signal does not represent tissue density and conventional MR sequences cannot image bone. A novel zero echo time (ZTE) MR sequence has been previously developed which generates signal from cortical bone with images acquired in 65 s. This has been combined with a deep learning model to generate a synthetic computed tomography (sCT) for MR-only radiotherapy. This study aimed to evaluate this algorithm for PET-MR attenuation correction in the pelvis.

**Methods:**

Ten patients being treated with ano-rectal radiotherapy received a $^{18}$F-FDG-PET-MR in the radiotherapy position. Attenuation maps were generated from ZTE-based sCT (sCTAC) and the standard vendor-supplied MRAC. The radiotherapy planning CT scan was rigidly registered and cropped to generate a gold standard attenuation map (CTAC). PET images were reconstructed using each attenuation map and compared for standard uptake value (SUV) measurement, automatic thresholded gross tumour volume (GTV) delineation and GTV metabolic parameter measurement. The last was assessed for clinical equivalence to CTAC using two one-sided paired *t* tests with a significance level corrected for multiple testing of $p \le 0.05/7 = 0.007$. Equivalence margins of $\pm 3.5\%$ were used.

**Results:**

Mean whole-image SUV differences were −0.02% (sCTAC) compared to −3.0% (MRAC), with larger differences in the bone regions (−0.5% to −16.3%). There was no difference in thresholded GTVs, with Dice similarity coefficients $\ge 0.987$. However, there were larger differences in GTV metabolic parameters. Mean differences to CTAC in ${\mathrm {SUV}}_{\max}$ were $1.0 \pm 0.8\%$ (± standard error, sCTAC) and $-4.6 \pm 0.9\%$ (MRAC), and $1.0 \pm 0.7\%$ (sCTAC) and $-4.3 \pm 0.8\%$ (MRAC) in ${\mathrm {SUV}}_{\rm mean}$. The sCTAC was statistically equivalent to CTAC within a $\pm 3.5\%$ equivalence margin for ${\mathrm {SUV}}_{\max}$ and ${\mathrm {SUV}}_{\rm mean}$ ($p = 0.007$ and $p = 0.002$), whereas the MRAC was not ($p = 0.88$ and $p = 0.83$).

**Conclusion:**

Attenuation correction using this radiotherapy ZTE-based sCT algorithm was substantially more accurate than current MRAC methods with only a 40 s increase in MR acquisition time. This did not impact tumour delineation but did significantly improve the accuracy of whole-image and tumour SUV measurements, which were clinically equivalent to CTAC. This suggests PET images reconstructed with sCTAC would enable accurate quantitative PET images to be acquired on a PET-MR scanner.

## Background

Simultaneous positron emission tomography–magnetic resonance (PET-MR) enables high-quality anatomic, functional and metabolic information to be acquired with high degrees of spatial alignment in the same imaging session [1]. This has potential benefits for improved staging and treatment response assessment in rectal cancer [2, 3] as well as improved radiotherapy gross tumour volume (GTV) delineation [4] and identification of active tumour sub-volumes for dose painting [5]. This utilises the superb soft tissue contrast of MR anatomical imaging, as well as its functional imaging ability such as diffusion-weighted (DW) MR [6]. However, this comes at the cost of accurate attenuation correction of the patient, compared to PET–computed tomography (CT).

Conventional MR images provide little signal from both low PET attenuating materials such as air and high PET attenuation materials such as cortical bone [7]. Therefore, there is no one-to-one map possible from conventional MR intensity values to linear attenuation coefficients that applies to the whole patient image, unlike for a CT scan [8]. The current vendor-supplied solution for the pelvis, MR attenuation correction (MRAC), utilises a Dixon MR sequence to segment air, lung, fat and soft tissue compartments, which are then assigned population values [9]. This, however, introduces PET attenuation errors through the omission of any bone information, with reported maximum standard uptake value (SUV) errors in soft tissue lesions of −6% and in bone lesions of −11% (estimated median relative differences from boxplot) [10].

This situation is very similar to the problem faced within MR-only radiotherapy, where MR cannot be used directly for radiotherapy dose calculations [11]. This has led to development of methods to generate a synthetic CT (sCT) from MR images which can then be used for dose calculations. Therefore, there is potential to apply these sCT algorithms to PET attenuation correction. This could facilitate a streamlined workflow with a single radiotherapy planning PET-MR examination as a ‘one-stop shop’ [12]. These algorithms have been designed and validated for megavoltage radiotherapy dose calculations, which in principle is less stringent than PET attenuation correction because of the higher photon energy in radiotherapy beams.

Two previous studies have investigated applying radiotherapy sCT algorithms for PET attenuation correction in the pelvis. Wallstén et al. used an atlas-based sCT derived from T2-weighted MR images for PET attenuation correction in 12 prostate cancer patients [13]. They reported reduced mean SUV differences to CT in bone regions which translated into significantly reduced SUV differences in the PET-avid prostate sub-volume ($p<0.001$). Ahangari et al. evaluated a deep learning sCT algorithm based on the Dixon MR sequence for cervix radiotherapy patients [12]. The model was trained with 26 patients and evaluated on seven, with small mean differences in tumour SUVs to CT ($<1.0\%$).

There have also been a number of studies investigating methods exclusively developed for attenuation correction for PET-MR in the pelvis, including methods based on deep learning [14]. Bradshaw et al. used diagnostic quality T1 and T2 MR images as inputs for a deep learning algorithm to produce a four-compartment (air, fat, water and bone) sCT trained on 12 patients [15]. Differences in ${\mathrm {SUV}}_{\max}$ of soft tissue lesion to reference CT attenuation correction (CTAC) were $-1.0 \pm 1.3\%$. Abrahamsen et al. used a deep learning model based on Dixon MR images trained with 22 patients for prostate-specific membrane antigen (PSMA) PET [16]. Median absolute percentage differences in ${\mathrm {SUV}}_{\max}$ compared to the CTAC for 17 test patients were 3.8% and 2.2% in bone and soft tissue lesions, respectively. Another Dixon-based deep learning algorithm for PSMA-PET was developed by Pozaruk et al. using images from 18 patients [17]. They reported mean absolute differences within the prostate for 9 test patients of $0.75 \pm 0.52\%$ and $0.64 \pm 0.62\%$ for ${\mathrm {SUV}}_{\max}$ and ${\mathrm {SUV}}_{\rm mean}$, respectively.

Another radiotherapy sCT algorithm has been developed based on a zero echo time (ZTE) imaging sequence, which provides MR signal from bone [18]. Since this is the primary deficiency in the current MRAC technique, ZTE-based sCT algorithms potentially could improve PET attenuation correction significantly [10]. However, to the best of the authors’ knowledge, no study has investigated applying a ZTE-only based radiotherapy sCT for PET attenuation correction in ano-rectal cancer patients. The aim of this study was to apply a ZTE-based deep learning sCT algorithm developed for pelvic MR-only radiotherapy dose calculations to PET-MR attenuation correction for ano-rectal cancer patients. Since the aim was to evaluate the equivalence in PET-MR attenuation correction between sCT and CT, the statistical analysis carried out would not be conventional superiority testing but equivalence testing [19]. This statistical approach has been applied in the MR-only radiotherapy literature [20] but has not been used previously for PET-MR attenuation correction analysis.

## Materials and methods

### Patient data collection

The study population consisted of 10 patients (four male and six female) who were all enrolled in the deep MR-only RT study (research ethics committee reference 20/LO/0583) and received a PET-MR scan. Patients were diagnosed with anal cancer (*n* = 6) stages T1/2N0M0-T2N1M0 and rectal cancer (*n* = 4) stages T2N0M0-T3b/T4N0M0 and had a median age of 65 years (range 49-76). All patients were planned for radical/neoadjuvant chemoradiotherapy. Patients were excluded if they were contraindicated for MR scanning, had medical implants in the pelvic area (e.g. hip prostheses), were unable to fit inside the coil bridge or were unable to fast for 6 h.

All patients received a simultaneous PET-MR scan on a SIGNA PET/MR 3T scanner (version MP26 GE Healthcare, Waukesha, USA) after their radiotherapy planning CT scan and before their first treatment fraction. Patients were scanned in the radiotherapy treatment position on a flat couch top with a coil bridge supporting the anterior MR coil and a combined customisable foot and knee rest (Civco), with their position adjusted to match external lasers to the radiotherapy patient tattoos. All patients had fasted for 6 h prior to injection and had a measured blood glucose concentration of $< 10\;\mathrm {mmol\,L^{-1}}$. Patients were injected with $3.5\;\mathrm {MBq\,kg^{-1}}\pm 10\%$ of $\mathrm {^{18}F\text{- }}$fluorodeoxyglucose (FDG) (one patient received $1.7\;\mathrm {MBq\,kg^{-1}}$), with PET images starting to be acquired 73 min (median, range 60-86 min) post-injection. The PET acquisition consisted of one 5 min bed position with the patient tumour centred in the PET field of view. Images were reconstructed using an ordered subset expectation maximisation (OSEM) algorithm with 4 iterations and 16 subsets and a $5.0\;\textrm{mm}$ Gaussian filter using the manufacturer provided offline reconstruction tool Duetto (version 2.17, GE Healthcare) in MATLAB (version 2017a, MathWorks, Natick, Massachusetts, USA). Point spread function correction and time of flight information were utilised. Images were reconstructed with a $60 \times 60\;\mathrm {cm^2}$ axial field of view, a $256 \times 256$ axial matrix and 89 slices with a slice thickness of $2.78\;\textrm{mm}$.

Two MR sequences were acquired: a novel ZTE sequence [21] and the standard Dixon sequence used for the scanner-generated PET attenuation correction maps. The ZTE sequence was acquired with flip angle $1^\circ$, nominal field of view $360 \times 360 \times 300\;\mathrm {mm^3}$, resolution $2.0 \times 2.0 \times 2.0\;\mathrm {mm^3}$, repetition time $TR=1.06\;\textrm{ms}$, nominal echo time $TE=0.016\;\textrm{ms}$, and 59392 3D centre-out radial spokes. Chemical shift was minimised by adjusting the centre frequency to be between fat and water [22] and using a receive bandwidth of $694\;\mathrm {Hz\,pixel^{-1}}$. The duration of the sequence was 65 s and the acquisition started 29 min after the PET acquisition started (median, range 27-37 min). Image reconstruction was based on 3D gridding, including two-fold field of view extension to $720 \times 720 \times 600\;\mathrm {mm^3}$ (enabled by two-fold radial oversampling), deep learning-based de-noising and de-ringing [23] and 3D geometry correction.

The Dixon sequence was the automatic sequence used to generate the MRAC map. It had a voxel size of $2.0 \times 2.0 \times 5.2\;\mathrm {mm^3}$, with $2.6\;\textrm{mm}$ slice gaps, a field of view $500\times 500\times 312 \;\mathrm {mm^3}$, a repetition time $TR=4.05\;\textrm{ms}$, echo times $TE=2.232\;\textrm{ms}$ (in-phase) and $TE=1.116\;\textrm{ms}$ (out-phase) and a received bandwidth of $1302\;\mathrm {Hz\, pixel^{-1}}$. It had an acquisition duration of 14.8 seconds and occurred concurrently with the start of the PET acquisition. In addition, the scanner geometric accuracy and PET SUV accuracy were tested monthly during a radiotherapy quality assurance programme [24].

All patients received contrast-enhanced CT scans (Sensation Open, Siemens, Erlangen, Germany) in the radiotherapy planning positon with the same design of foot and knee rest and tattoo marks matched to external lasers. Images were acquired with a voxel size of $1.1\times 1.1\times 3\;\mathrm {mm^3}$ and a tube voltage of $V=120\;\textrm{kVp}$. CT images were acquired within 6 days (median, range 5-13 days) of the PET-MR scan.

### Synthetic CT generation

The sCT was generated by a deep learning algorithm from the ZTE image developed by GE Healthcare for MR-only radiotherapy. The algorithm as described previously [18, 25], consisted of a 2D convolution neural network adapted to a multi-task UNet framework. The network is trained by multiple loss functions, each designed to focus specifically on image translation, bone segmentation, and bone density value estimation. This supervised learning model was trained on co-registered pairs of ZTE and CT images from 36 pelvic radiotherapy patients (28 for model training and 8 for validation), which did not include the 10 patients in this study. The training data was augmented six-fold using random flips, zoom-in and zoom-out, in-plane and 3D rotation and the addition of Gaussian noise. The sCT algorithm was applied to PET attenuation correction without modification.

### Attenuation correction maps

PET images were reconstructed with three different attenuation correction maps for each patient. All attenuation correction maps included the coil components within the scanner bed, the radiotherapy couch, coil bridge and anterior coil as described previously [26, 27] and a model of the patient. The patient model varied between the different maps. The gold standard patient model (CTAC) consisted of the patient CT rigidly registered to the in-phase MR image in RayStation (v9B, RaySearch Laboratories, Stockholm, Sweden). A rigid registration was appropriate because the patient position was the same between images due to both being acquired in the radiotherapy position. Each patient registration was reviewed to ensure that the patient alignment was sufficient. Small differences in external contour were removed by cropping the registered CT to the in-phase MR external contour, with any missing tissue set to water density. Differences between images in the position of air pockets within the patient were removed by automatically delineating them on the CT and setting them to water density. The CT was converted to $511\;\textrm{keV}$ linear attenuation coefficient map using the PET-MR vendor-supplied calibration curve (GE Healthcare).

A second map was generated using the sCT image (sCTAC). Although the sCT was derived from the ZTE image acquired in the same scanning session as the in-phase MR, it was acquired 29 min later (median, range 25-37 min). Therefore, in case of patient motion, the sCT was rigidly registered to the in-phase MR image in RayStation and cropped to the in-phase MR external contour, with any missing tissue set to water density. The sCT automatically converted air pockets within the patient to water density. This was converted to $511\;\textrm{keV}$ linear attenuation coefficient map using the same calibration curve.

**Fig. 1:**
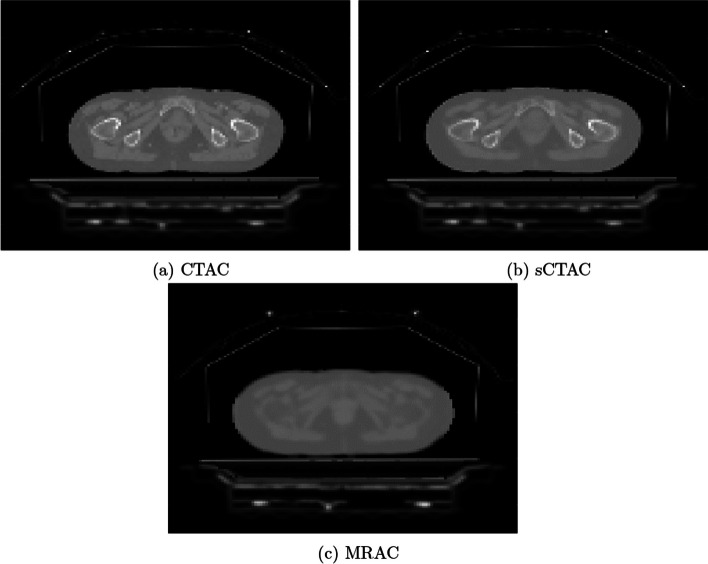
Attenuation correction maps for an example patient. All maps included the MR coils components, flat couch top and coil bridge. Patient models were based on CT (**a**), ZTE-derived sCT (**b**) and Dixon derived MR (**c**)

The final map used the standard vendor-supplied (GE Healthcare) patient model derived from the automatic Dixon sequence (MRAC). This method segmented the MR into four tissue classes: air, lung, fat and soft tissue, and assigned population-derived bulk density $511\;\textrm{keV}$ linear attenuation coefficients to each class [9]. Air pockets within the patient were converted to water density automatically. Despite the MRAC being derived from the in-phase MR, the MRAC contour was slightly larger due to differences in image resolution between the DIXON and MRAC. Therefore, the MRAC was also cropped to the in-phase MR external contour to ensure all attenuation correction maps had the same external contour. Examples of the three attenuation maps are shown in Fig. 1.

### Tumour delineation

**Fig. 2:**
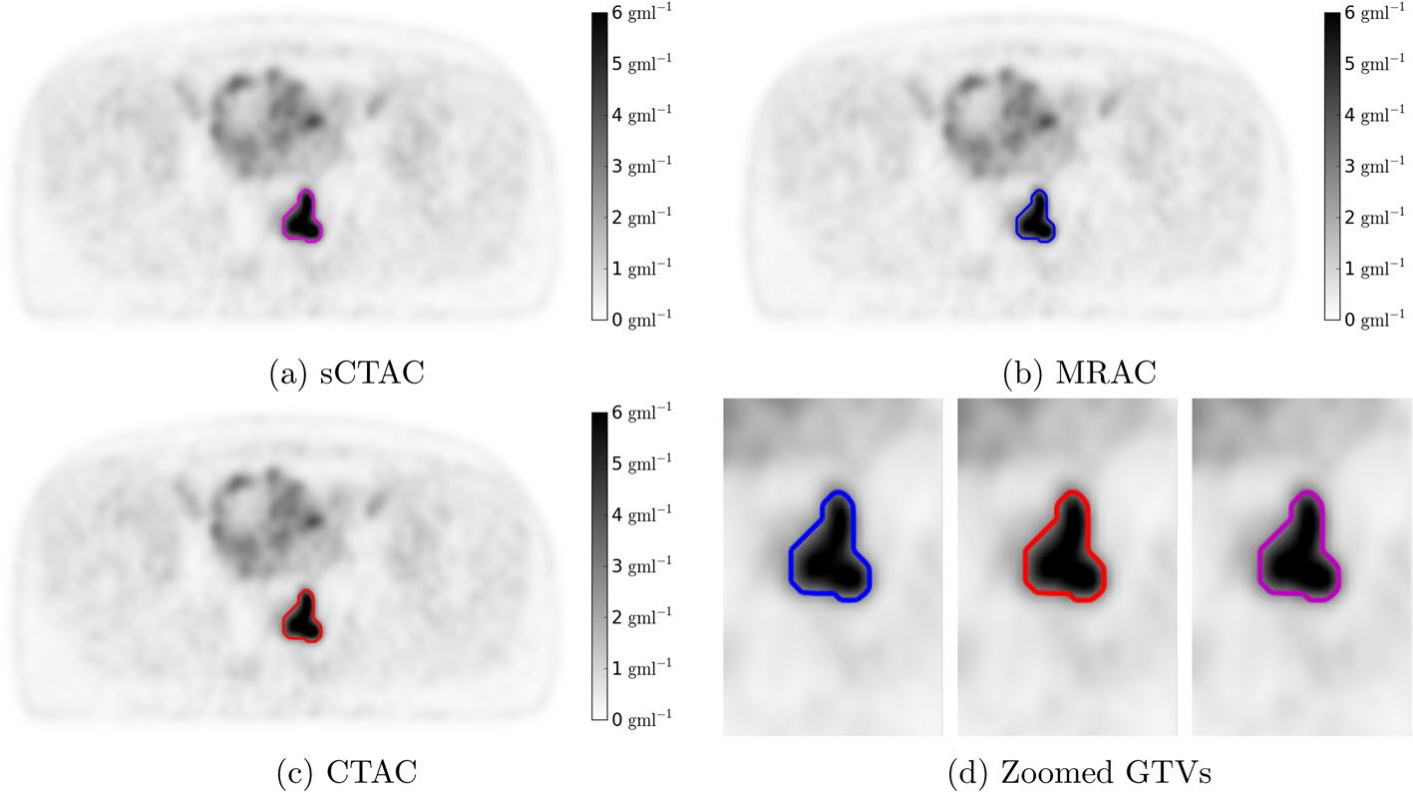
Example PET images reconstructed using the sCTAC (**a**), MRAC (**b**) and CTAC (**c**) attenuation correction maps. The threshold GTV contour is shown in purple, blue and red, respectively. Zoomed in pictures of the same GTVs are shown in (**d**). The patient was selected as having the sCTAC and MRAC ${\mathrm {SUV}}_{\max}$ differences closest to the mean differences

GTVs were automatically thresholded on each PET image using 40% of the maximum SUV [28] within a manual tumour volume contoured by an experienced consultant PET radiologist using RayStation. Primary and nodal volumes were delineated separately. Examples of the three PET images and automatic GTVs are shown in Fig. 2.

### Data analysis

**Fig. 3:**
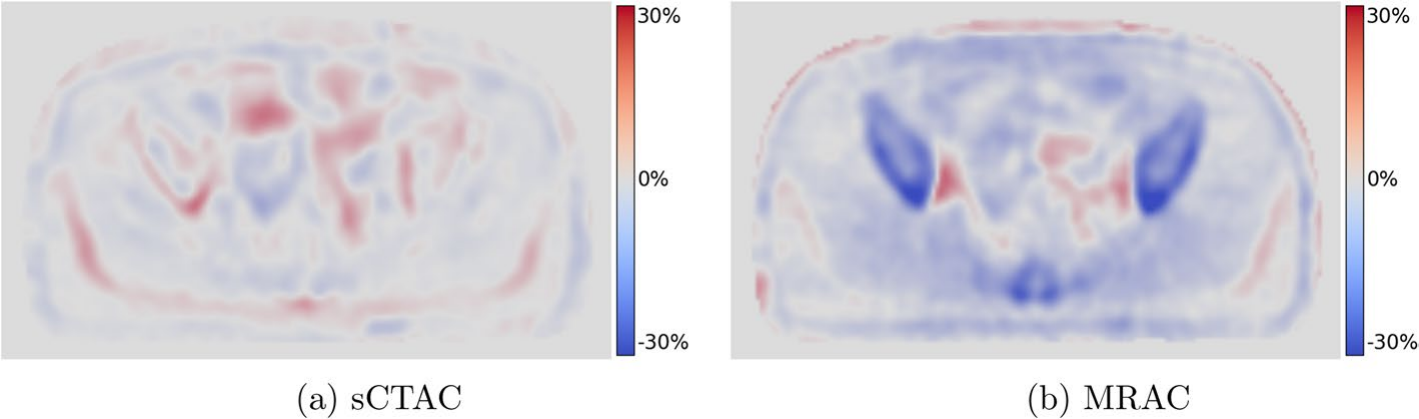
Example SUV difference maps to CTAC PET images for the sCTAC PET image (**a**) and MRAC PET image (**b**) for the same patient and slice as shown in Fig. 2

The per pixel percentage difference in SUV for MRAC - CTAC and sCTAC - CTAC relative to CTAC was calculated using MICE toolkit (v2021.2.1) [29]. Only differences within the CTAC external contour were included. This was automatically contoured using a threshold of $0.05\;\mathrm {gml^{-1}}$, and the same contour applied to the sCTAC and MRAC images. Relative SUV differences were binned into 400 bins between −100% and +100% for each patient, and the mean difference within each bin over all patients determined. An example whole-image difference map is shown in Fig. 3.

A major discrepancy between the MRAC and gold standard CTAC is that the MRAC does not reproduce bone. Therefore, SUV differences in the bone region were additionally investigated. A bone region of interest was automatically delineated on the CT using the ‘Bone ROI’ function in RayStation. This uses thresholding and connected regions function to contour bone. The per pixel percentage difference in SUV was calculated as described above but only within the region masked by the bone contour.

The similarity of the automatic GTV contours on sCTAC and MRAC PET images to CTAC was determined using the volumetric Dice similarity coefficient (DSC), the mean and maximum distances to agreement and the GTV volume, all calculated within RayStation. The accuracy of the calculation of a set of metabolic parameters on the sCTAC and MRAC GTVs was determined by comparing to CTAC measurements. The metabolic parameters assessed were ${\mathrm {SUV}}_{\max}$ and ${\mathrm {SUV}}_{\rm mean}$ of the tumour volumes. Large variation between patient SUVs meant the volume and metabolic comparisons between sCTAC and MRAC with CTAC were carried out as per-patient percentage differences relative to CTAC.

The SUV results were statistically tested for equivalence, with a null hypothesis that the sCTAC/MRAC PET images were different to the CTAC images. This is the opposite to conventional superiority testing which aims to determine if differences are statistically significant and has a null hypothesis that the sCTAC/MRAC PET images are not different to CTAC images. Equivalence between MRAC/sCTAC and CTAC was assessed using two one-sided t tests for paired data [19]. Tests were done for differences in ${\mathrm {SUV}}_{\max}$ and ${\mathrm {SUV}}_{\rm mean}$, for both MRAC and sCTAC and both primary and nodal GTVs (i.e. 8 tests in all). A significance level of $p \le 0.05$ was used, corrected for multiple testing by $p < 0.05/(8 - 1) = 0.007$ [30].

Equivalence testing considers sCTAC/MRAC PET images clinically equivalent to CTAC images if the SUV differences are smaller than a pre-defined equivalence margin, which is the maximum difference that would be considered clinically unimportant. There were no reported equivalence margins for PET attenuation correction in the literature. Therefore, an equivalence margin was defined as the maximum difference that would not increase the overall literature PET-CT SUV uncertainty by $> 0.5\%$. The method assumed that the only additional SUV uncertainty from PET-MR compared to PET-CT was due to attenuation correction, which was independent of all other PET uncertainties. Therefore, the attenuation correction uncertainty can be added in quadrature:

1 $$\begin{aligned} \Delta _{\rm PETMR} = \sqrt{\Delta _{\rm PETCT}^2 + \Delta _{\rm AC}^2}, \end{aligned}$$

where $\Delta _{\rm PETMR}$ is the overall PET-MR SUV uncertainty, $\Delta _{\rm PETCT}$ is the overall PET-CT uncertainty and $\Delta _{\rm AC}$ is the attenuation correction uncertainty. The equivalence margin ($\Delta _{\rm AC}$) was defined such that $\Delta _{\rm PETMR} - \Delta _{\rm PETCT} \le 0.5\%$. Literature values for PET-CT SUV repeatability were taken as 12%, using a meta-analysis of repeatability tests on the same scanner [31]. Using $\Delta _{\rm PETCT} = 12\%$ in equation (1) gives an equivalence margin of $\Delta _{\rm AC} = 3.5\%$.

## Results

sCTs were successfully generated for each patient. There were 9 primary and 5 nodal GTVs contoured, (one patient had no primary following surgery before chemoradiotherapy).

**Fig. 4:**
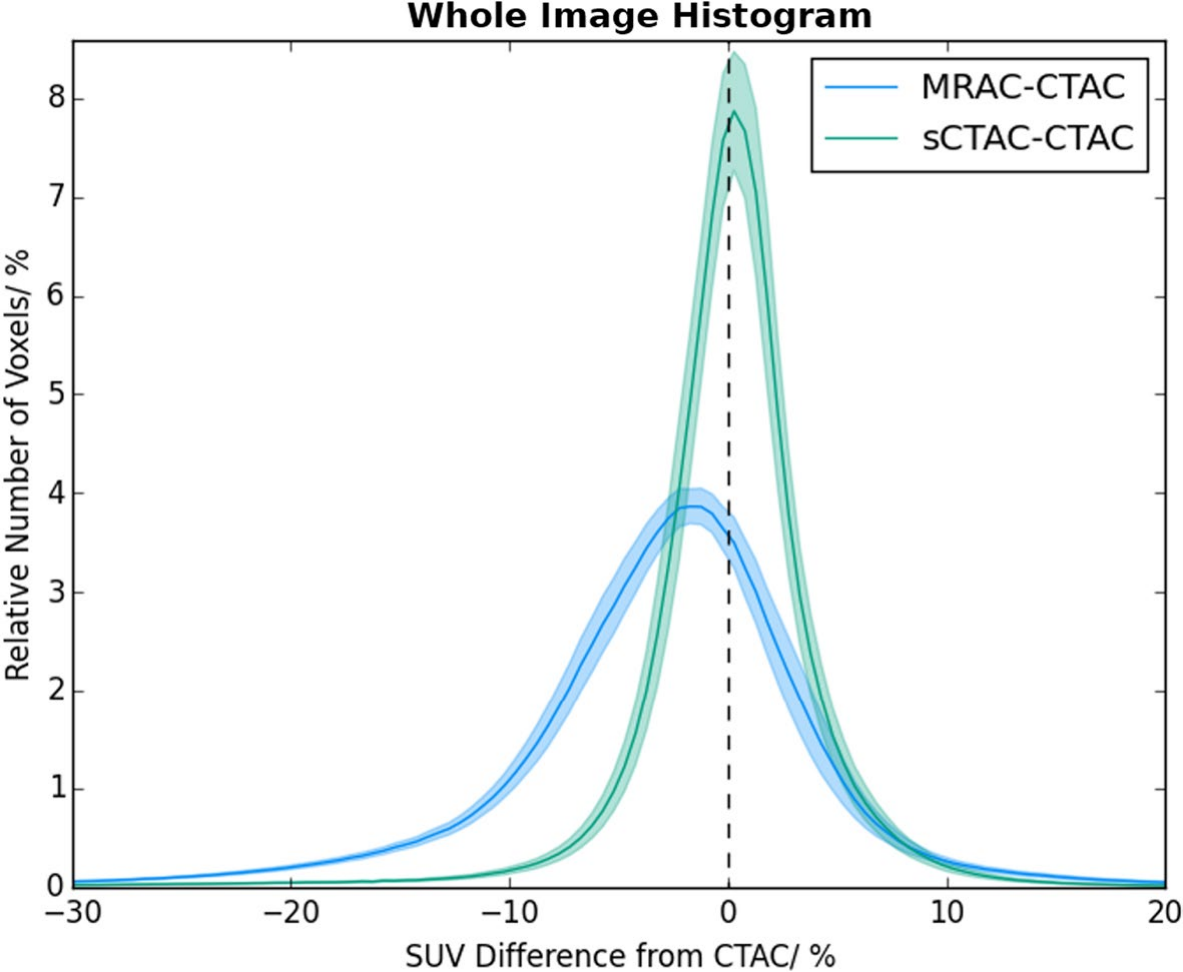
Histogram of relative number of voxels with percentage differences in SUV to CTACT for sCTAC (green) and MRAC (blue). Relative number of voxels given as percentage of total voxels within patient external contour. Solid lines show mean counts over all patients for each bin, and shaded areas ± one standard error. The dashed line indicates zero difference

**Fig. 5:**
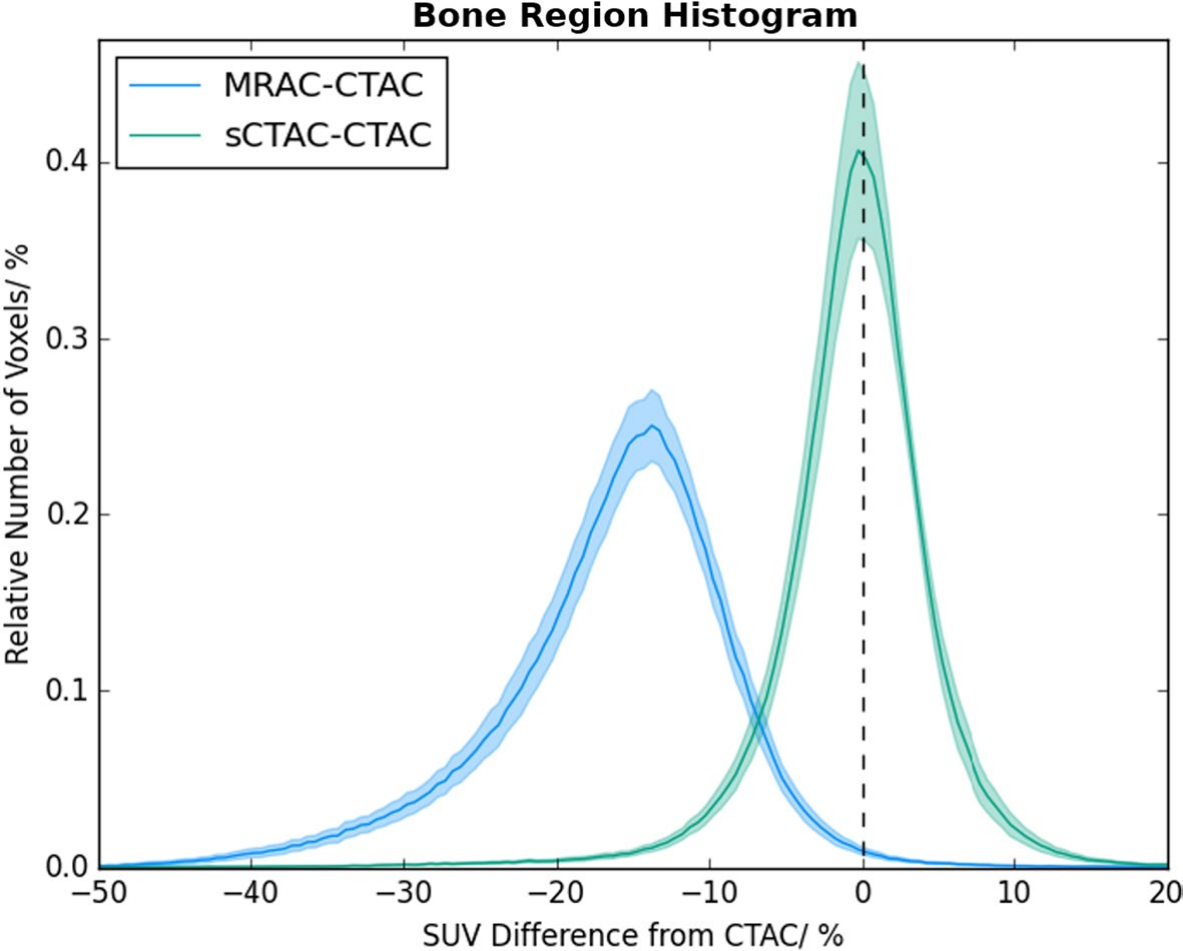
Histogram of relative number of voxels with percentage differences in SUV to CTAC for sCTAC (green) and MRAC (blue) within the bone region. Relative number of voxels given as percentage of total voxels within patient external contour. Therefore y-axis is in the same units as Fig. 4 but the scale is different. Solid lines show mean counts over all patients for each bin, and shaded areas ± one standard error. The dashed line indicates zero difference

The whole-image SUVs in the sCTAC- and MRAC-reconstructed PET images were lower than those in the CTAC PET images with the mean difference being −3.0% for the MRAC and −0.02% for the sCTAC. The distributions of SUV differences were quite different, with the sCTAC SUV differences a much narrower distribution as well as closer to zero (see Fig. 4). The differences within the bone mask were much larger than the whole image, with the mean MRAC difference being −16.3% and the sCTAC difference −0.5% (see Fig. 5).

The thresholded GTVs for the primary and nodal tumours were very similar to CTAC on both MRAC and sCTAC, with all metrics agreeing within one standard error (Table 1). Both MRAC and sCTAC volume differences were close to zero (within two standard errors).

**Table 1:** Delineation metrics for GTVs on MRAC and sCTAC

**Metric**	**GTV**	**MRAC**	**sCTAC**
DSC	Primary	$0.990 \pm 0.002$(0.978, 0.994)	$0.992 \pm 0.002$(0.983, 0.998)
${\mathrm {DTA}}_{\rm mean}$[mm]	Primary	$0.06 \pm 0.01$(0.02, 0.13)	$0.06 \pm 0.01$(0.01, 0.11)
${\mathrm {DTA}}_{\max}$[mm]	Primary	$1.87 \pm 0.24$(0.97 ,3.16)	$1.76 \pm 0.13$(1.23, 2.53)
Volume [$\%$]	Primary	$0.7 \pm 0.5$($-0.4$, 4.2)	$0.7 \pm 0.5$($-1.3$, 3.2)
DSC	Nodal	$0.988 \pm 0.006$(0.968, 1.000)	$0.987 \pm 0.008$(0.955, 1.000)
${\mathrm {DTA}}_{mean}$[mm]	Nodal	$0.04 \pm 0.01$(0.00, 0.07)	$0.04 \pm 0.02$(0.0, 0.11)
${\mathrm {DTA}}_{\max}$[mm]	Nodal	$1.4 \pm 0.4$(0.0, 2.34)	$1.4 \pm 0.4$(0.0, 2.0)
Volume [$\%$]	Nodal	$0.8 \pm 0.3$(0.0, 1.6)	$1.1 \pm 0.6$($-0.1$, 2.8)

Volume indicates the volume difference between MRAC/sCTAC and CTAC, relative to the CTAC volume. All results given as mean ± standard error (minimum, maximum)

**Fig. 6:**
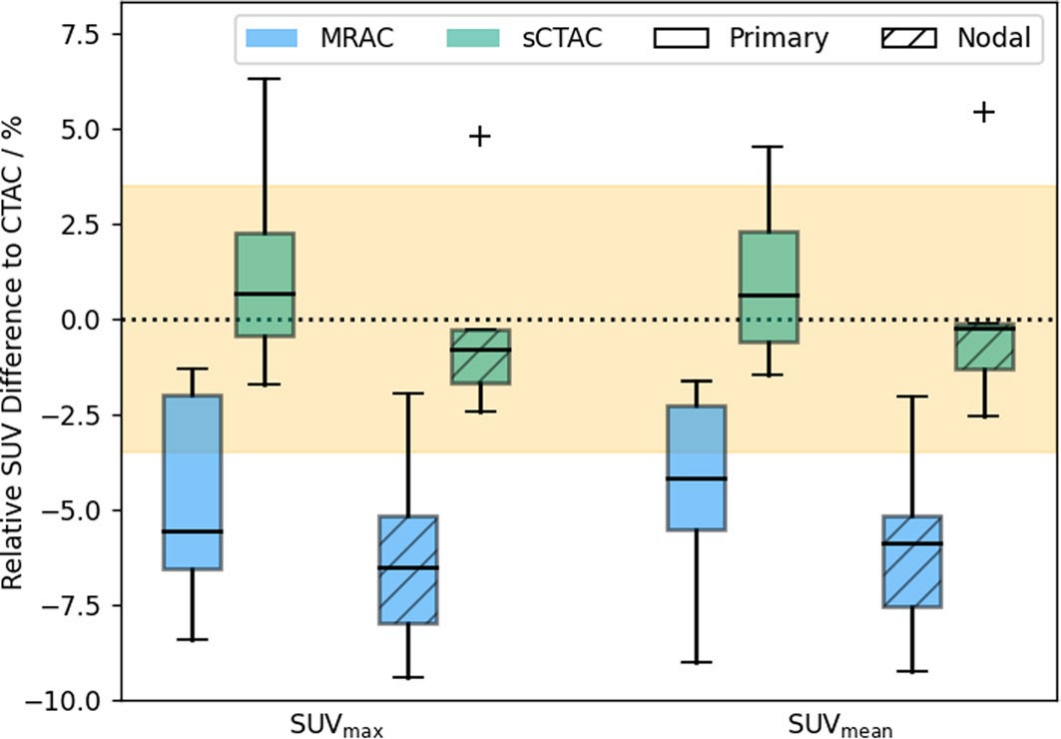
Boxplot of SUV differences to CTAC PET images for the MRAC (blue) and sCTAC (green) images. Solid bars indicate primary volumes (*n* = 9) and hatched bars nodal volumes (*n* = 5). The rectangles indicate the interquartile range (IQR), with the horizontal black line the median value, the black whiskers the maximum (minimum) data point within $Q3 + 1.5IQR$ ($Q1 - 1.5IQR$), and the black crosses outlier data points. The dotted line indicates zero difference, and the yellow filled regions indicate the equivalence margins ($\pm 3.5\%$)

There were larger differences between MRAC and sCTAC in the metabolic parameters. In the primary tumours, the mean MRAC differences in SUV from CTAC were $-4.6 \pm 0.9\%$ (± standard error, range −8.4%, −1.3%) for ${\mathrm {SUV}}_{\max}$ and $-4.3 \pm 0.8\%$ (−9.0%, −1.6%) for ${\mathrm {SUV}}_{\rm mean}$. The sCTAC SUV differences were closer to zero and less dispersed (Fig. 6), with differences of $1.0 \pm 0.8\%$ (−1.7%, 6.3%, ${\mathrm {SUV}}_{\max}$) and $1.0 \pm 0.7\%$ (−1.5%, 4.5%, ${\mathrm {SUV}}_{\rm mean}$). The absolute differences in SUV for the sCTAC were $1.8 \pm 0.6\%$ (0.1%, 6.3%, ${\mathrm {SUV}}_{\max}$) and $1.7 \pm 0.5\%$ (0.1%, 4.5%, ${\mathrm {SUV}}_{\rm mean}$). The MRAC was not clinically equivalent to CTAC within $\pm 3.5\%$ for ${\mathrm {SUV}}_{\max}$ ($p = 0.88$) or ${\mathrm {SUV}}_{\rm mean}$ ($p=0.83$). Conversely, the sCTAC was clinically equivalent to CTAC for both ${\mathrm {SUV}}_{\max}$ ($p = 0.007$) and ${\mathrm {SUV}}_{\rm mean}$ ($p = 0.002$).

The SUV differences between MRAC and sCTAC were greater for the nodal volumes than the primary tumours. ${\mathrm {SUV}}_{\max}$ differences were $-6.2 \pm 1.3\%$ ($-9.4\%$, $-1.9\%$, MRAC) and $-0.1 \pm 1.3\%$ (−2.4%, 4.8%, sCTAC) and ${\mathrm {SUV}}_{\rm mean}$ differences were $-6.0 \pm 1.2\%$ ($-9.2\%$, $-2.0\%$, MRAC) and $0.2 \pm 1.4\%$ ($-2.5\%$, $5.4\%$, sCTAC). Neither MRAC or sCTAC was clinically equivalent for ${\mathrm {SUV}}_{\max}$ ($p = 0.95$ and $p=0.03$, respectively), or ${\mathrm {SUV}}_{\rm mean}$ ($p=0.94$ and $p=0.04$). The absolute differences in nodal tumours SUV for the sCTAC were $2.0 \pm 0.8\%$ ($0.3\%$, $4.8\%$, ${\mathrm {SUV}}_{\max}$) and $1.9 \pm 1.0\%$ ($0.1\%$, $5.4\%$, ${\mathrm {SUV}}_{\rm mean}$).

## Discussion

This study has assessed the use of a MR-only radiotherapy sCT algorithm for PET-MR attenuation correction in the pelvis compared to the current Dixon-based MRAC. The sCTAC reduced the whole-image SUV difference to CTAC to −0.02%, compared to −3.0% for the MRAC. This did not translate into improvements in thresholded GTV delineation, with both MRAC and sCTAC having $DSC \ge 0.987$ for primary and nodal GTVs. However, differences in GTV metabolic parameters were larger, with differences to CTAC in ${\mathrm {SUV}}_{\max}$ being $1.0 \pm 0.8\%$ (sCTAC) rather than $-4.6 \pm 0.9\%$ (MRAC) for primary GTVs. ${\mathrm {SUV}}_{\rm mean}$ calculated on the sCTAC was clinically equivalent to CTAC values within a $\pm 3.5\%$ equivalence margin for primary GTVs with $p=0.002$, whereas the MRAC was not ($p=0.88$).

The sCTAC-reconstructed PET images had smaller SUV differences to CTAC over the whole image than the MRAC-reconstructed images (Fig. 4). The mean sCTAC difference was very close to zero, and the distribution of differences tightly clustered around the mean. In contrast, the MRAC SUV difference was larger (−3.0%) and had a substantially broader distribution of differences. The MRAC had substantially larger differences within the bone mask (−16.3% compared to −0.5%). Although these only made up a small proportion of the overall voxels in the image, the absence of bone also caused the MRAC to over-estimate SUVs adjacent to the bone (Fig. 3). This suggests that the ZTE-based sCT model is accurately capturing the bone information.

The automatic GTV delineation using thresholds of 40% of ${\mathrm {SUV}}_{\max}$ were very similar between CTAC and both sCTAC and MRAC. The DSC results ($\ge 0.987$, with 1.0 indicating perfect agreement) were higher than the reported inter-observer variability of 0.96 in automated rectal cancer GTV delineation on the same PET image [32]. This implies both sCTAC and MRAC provide sufficient SUV accuracy for accurate GTV delineation.

The differences in patient attenuation produced larger differences in the metabolic parameter analysis. The sCTAC SUV differences to CTAC were smaller than MRAC for both ${\mathrm {SUV}}_{\max}$ and ${\mathrm {SUV}}_{\rm mean}$ for both primary and nodal GTVs, and sCTAC was clinically equivalent to CTAC for the primary GTVS (within $\pm 3.5\%$), whereas the MRAC SUVs were not. The nodal GTVs were not clinically equivalent for either sCTAC or MRAC, although for the sCTAC this is likely due to there only being five patients having nodal GTV and the results from one patient that may have been an outlier (see Fig. 6). Examining the sCT and CT for this patient showed there was a small discrepancy in the bone alignment on the few slices that the nodal GTV was on (it only had a volume of $1.4\;\mathrm {cm^3}$). This resulted in more bone on the sCT for these slices than on the CT, resulting in an over-correction of the attenuation, leading to higher SUVs in the sCTAC image.

This highlights a limitation of this study which is that the CT image used as the gold standard was acquired on a different scanner and on a different day to the PET-MR image. Both CT and PET-MR images were acquired in the same radiotherapy position with the same immobilisation, each patient registration was reviewed to ensure it was appropriate, the external contours of all attenuation correction maps were cropped to be the same, and air pockets within the patient (which varied between images) were all set to water density. These all ensured a high degree of alignment between the images. However, there could still be small discrepancies between patient images, which would be confounding differences due to misalignments rather than incorrect HU assignment in the sCT. This could potentially be improved through the use of a deformable registration between CT and MR, although this would not completely remove discrepancies and may have introduced additional ones. PET-CT also suffers from this problem to some extent, where although the PET and CT images are acquired in the same imaging session, they are separated in time. This can result in discrepancies due to gross patient motion or changes in internal anatomy (e.g. from breathing).

These results demonstrate that the sCTAC-reconstructed PET images produce SUV differences that are clinically equivalent to CTAC for primary GTVs within equivalence margins of $\pm 3.5\%$ at the 90% confidence level (using a multiple testing corrected *p*-value of $p=0.05/(8-1)=0.007$ [30]). Equivalence testing is a well-established statistical methodology in clinical trials [33], although has not been used previously in evaluating PET-MR attenuation correction accuracy. Therefore, there was not a standard equivalence margin in the literature, which is critical to the validity of equivalence testing [19]. A methodology was developed to derive the margins of $\pm 3.5\%$. This assumed that the attenuation correction method is independent of the other uncertainties in the PET imaging and that increases in the overall SUV uncertainty from 12 to 12.5% can be considered negligible. The overall PET-CT uncertainty was estimated as the test–retest repeatability values of SUV measurements reported in the literature. Different values have been given, with a meta-analysis of five studies investigating the repeatability of PET-CT scans reporting values of 30% (${\mathrm {SUV}}_{\max}$) and 20% (${\mathrm {SUV}}_{\rm mean}$) [34]. However, this included data from patients scanned on different scanners which would increase variability in SUV measurements. A more recent meta-analysis of repeatability studies using data from patients scanned on the same scanner sequentially reported average repeatability of $\sim 10\%$, with a majority of studies reporting repeatability of $\le 12\%$ [31]. This is similar to the data reported in a study focused on SUV measurements in ano-rectal tumours, which reported repeatability of 10–12% [35]. This lead to the choice of 12% as the overall PET-CT uncertainty, from which was derived the $\pm 3.5\%$ equivalence margin.

The sCTAC results in this study compare well with previous published results on PET-MR attenuation correction in the pelvis. Shandiz et al. investigated using a short echo time sequence and automated image segmentation techniques to generate a bulk density sCT with five tissue classes (cortical bone, air cavity, fat, soft tissue and background) [36]. PET errors were estimated using simulated PET data for one healthy patient, with mean voxel-by-voxel SUV errors of $-14 \pm 15\%$, $4 \pm 6\%$, $8\pm 13\%$ and $4\pm 2\%$ in the bone, soft tissue, fat and prostate regions. Bradshaw et al. used a deep learning model to generate a four tissue class sCT from T1 and T2 Dixon MR images from 12 patients [15]. This was evaluated on 16 FDG-avid lesions from five patients, with mean ${\mathrm {SUV}}_{\max}$ differences of $-1.0 \pm 1.3 \%$, the same magnitude of difference reported in this study ($1.0 \pm 0.8\%$). The same ZTE sequence investigated here was used in combination with a Dixon MR images in a deep learning model with 10 training patients to generate sCTs [10]. Median ${\mathrm {SUV}}_{\max}$ differences for 30 bone lesions from 16 patients were −1% (range −8%, 3%, relative differences estimated from boxplot) and −2% (−12%, 5%) for 60 soft tissue lesions. Two other studies developed deep learning models for prostate lesions using PSMA-PET. Abrahamsen et al. reported median absolute percentage differences to CTAC in ${\mathrm {SUV}}_{\max}$ of 2.2% for soft tissue lesions [16], very similar to the $1.8 \pm 0.6\%$ and $2.0 \pm 0.8\%$ absolute differences for the primary and nodal tumours in this study. Pozaruk et al. found slightly smaller absolute differences of $0.75 \pm 0.52\%$ and $0.64 \pm 0.62\%$ for ${\mathrm {SUV}}_{\max}$ and ${\mathrm {SUV}}_{\rm mean}$, respectively [17]. However, these measurements were restricted to the prostate organ and so may not be not directly comparable to the results from rectal primary and nodal tumours in this study.

The results in this study also demonstrated comparable or superior performance to other MR-only radiotherapy sCT algorithms which had been applied to PET attenuation correction. Wallstén et al. gave whole-image SUV differences of −0.5% and within-bone differences of −4.2% [13], which were larger than the −0.02% and −0.5% reported here. This translated into ${\mathrm {SUV}}_{\rm mean}$ differences in PET-avid lesions within the prostate of −2.3%, again a larger difference than the $1.0 \pm 0.7\%$ reported in this study. Ahangari et al. found mean differences in ${\mathrm {SUV}}_{\max}$ of $-0.8 \pm 1.2 \%$ (± standard error, range −4.9%, 4.7%, estimated from bar graph) and in ${\mathrm {SUV}}_{\rm mean}$ of $-0.3 \pm 1.8 \%$ ($-5.9\%$, $7.4\%$) [12]. These were similar to the results reported here (absolute differences $\le 1.0\%$).

The MRAC results also show good agreement with the literature. Wallstén et al. found mean SUV differences within soft tissue of $-3.6\%$ and within the bone region of $-17.7\%$ [13], very similar to the $-3.0\%$ and $-16.3\%$ found here. The ${\mathrm {SUV}}_{\rm mean}$ difference in the prostate PET-avid lesion was $-5.9\%$, similar to the $-4.3 \pm 0.8\%$ reported here. Leynes et al. found median ${\mathrm {SUV}}_{\max}$ differences in soft tissue lesions of −6% (−18%, 4%) [10], which agrees within two standard errors with the $-4.6 \pm 0.9\%$ (−8.4%, −1.3%) reported in this study.

There are two aspects to consider when applying radiotherapy sCT algorithms to PET attenuation correction. On the one hand, PET is more sensitive to HU errors due to the lower energy of PET photons compared to those produced by megavoltage linear accelerators, so this makes PET attenuation correction more challenging for sCT algorithms. On the other hand, the overall uncertainty of SUV measurements is much higher than in radiotherapy dosimetry. The overall repeatability of SUV measurements is 10–12% [31], whereas the overall uncertainty in radiotherapy dose delivered to the patient is 3–5% [37]. Thus, clinical equivalence in SUV accuracy could be achieved with differences of 3–4% whereas the dose uncertainty of any individual component of the radiotherapy pathway has to be $\le 1\%$ to not increase the overall dose uncertainty [37]. This suggests that sCT requirements for MR-only radiotherapy are more stringent than for PET attenuation correction, and so sCTs that are clinically acceptable for radiotherapy are likely to be able to be used for PET attenuation correction without modification. This agrees with the data found in this study and the two other studies applying radiotherapy developed sCTs to PET attenuation correction [12, 13].

A limitation of this study was that only small numbers of patients were evaluated, especially for the nodal evaluation. This is likely to have prevented clinical equivalence in nodal SUV measurements being demonstrated due to the study being under-powered. In addition, measurements have only been made on one manufacturer’s scanner in one centre, which was the same scanner on which the ZTE images used to the sCT model were acquired on. Evaluating the sCT algorithm on more patients acquired in different centres and on different scanners would enable the generalisability of this method to be tested [14].

## Conclusions

A ZTE-based deep learning sCT algorithm for MR-only radiotherapy has been successfully applied for PET-MR attenuation correction. There were substantial reductions in SUV differences to gold standard CTAC, with mean whole-image differences being $-0.02\%$, compared to $-3.0\%$ for the current MRAC. The improvements in the bone regions were particularly large, $-0.5\%$ rather than $-16.3\%$. This had no impact on the accuracy of thresholded GTV delineation. However, it did have a significant impact on metabolic parameters, with SUV differences in ${\mathrm {SUV}}_{\max}$ and ${\mathrm {SUV}}_{{\rm mean}}$ being $1.0 \pm 0.8\%$ and $1.0 \pm 0.7\%$, respectively, rather than $-4.6 \pm 0.9\%$ and $-4.3 \pm 0.8\%$ for the MRAC. The SUV measurements in the primary GTVs were clinically equivalent to CTAC within $\pm 3.5\%$, respectively ($p=0.007$ and $p=0.002$), whereas MRAC measurements were not ($p = 0.83$ and $p = 0.83$). This suggests that PET images reconstructed using sCTAC substantially improve in SUV accuracy compared to current MRAC approaches and were comparable to PET images reconstructed using other deep learning state-of-the-art attenuation correction methods. Using this sCT developed for MR-only radiotherapy without modification would enable highly accurate quantitative PET images in the pelvis to be acquired on a PET-MR scanner.

## Data Availability

The datasets used and/or analysed during the current study are available from the corresponding author on reasonable request.

## Abbreviations

PET: Positron emission tomography
MR: Magnetic resonance
GTV: Gross tumour volume
DW-MR: Diffusion-weighted MR
CT: Computed tomography
MRAC: MR attenuation correction
sCT: Synthetic CT
SUV: Standard uptake value
ZTE: Zero echo time
FDG: Fluorodeoxyglucose
OSEM: Ordered subset expectation maximisation
DSC: Dice similarity coefficient
